# Neuronal number and somal volume in calbindin-expressing neurons of the marmoset dorsal lateral geniculate nucleus are preserved during aging

**DOI:** 10.1371/journal.pone.0323906

**Published:** 2025-05-23

**Authors:** Nelyane N. M. Santana, Maria M. O. da Silva, Eryck H. A. Silva, Sâmarah F. dos Santos, Lyzandro L.F. Bezerra, Wellydo K. M. Escarião, Gabriel A. M. Vasiljevic, Felipe P. Fiuza, Jeferson S. Cavalcante, Rovena Clara Engelberth

**Affiliations:** 1 Department of Physiology and Behavior, Laboratory of Neurochemical Studies, Bioscience Center, Federal University of Rio Grande do Norte, Natal, Brazil; 2 Edmond and Lily Safra International Institute of Neuroscience, Santos Dumont Institute, Macaíba, Brazil; Children's Hospital Affiliated of Zhengzhou University: Zhengzhou Children's Hospital, CHINA

## Abstract

Compelling evidence links age-related brain dysfunction and neurodegenerative processes to persistent disruptions in intracellular calcium (Ca^2+^) signaling, a central hypothesis in the Ca^2+^ theory of aging. Calbindin (CB), a classical Ca^2+^ buffer, has been implicated in region-specific susceptibility to aging-related effects. Specifically, CB-immunopositive (CB^+^) neurons have demonstrated an age-dependent decline in neuronal number across various cortical and subcortical regions. However, it remains unclear whether this decrease occur in the dorsal lateral geniculate nucleus (DLG), a crucial relay and modulatory center for visual processing. Additionally, the potential impact of aging on the cellular volume of CB^+^ neurons in the DLG has not been fully elucidated, albeit an age-dependent neuronal hypertrophy of this region has been reported. To address these questions, we investigated CB^+^ neurons in the DLG of six marmosets (*Callithrix jacchus*), aged between 29–143 months. Using design-based stereological techniques, we estimated the total number and somal volume of CB^+^ neurons in DLG layers. Our results revealed no signs of CB^+^ neuronal number loss and somal volumetric changes in aged DLG, particularly within the koniocellular layers, a stratum that primarily expresses CB and play a critical role in blue/yellow color vision. Altogether, our findings suggest a preserved neuronal number and cellular volume of the CB^+^ population during aging process in the marmoset DLG. Moreover, they provide a valuable basis for future investigations into the neuroprotective role of CB in visual processing during aging and open avenues for strategies designed to preserve vulnerable neuronal populations in age-related neurodegenerative conditions.

## 1. Introduction

One of the most prominent and well-established theories in gerontology is the calcium (Ca^2+^) hypothesis of aging [1–3]. This theory posits that a sustained disruption in the regulatory mechanisms governing intracellular Ca^2+^ signaling contributes to the triggering of detrimental changes in neuronal function and survival, particularly in aging and age-related neurodegenerative diseases [4,5]. Specifically, alterations in the protein-mediated mechanisms of cytosolic Ca^2+^ buffering, including the action of the EF-hand domain family of Ca^2+^ binding proteins (CBPs), may result in intracellular dysregulation of this ion [6], notably through the sustained rise of Ca^2+^ levels [7]. This dyshomeostasis renders neurons selectively vulnerable to intracellular Ca^2+^ fluctuations, leading to a dysfunctional cellular profiles and degeneration through Ca^2+^-mediated toxicity [8].

Calbindin (CB), a member of the CBPs, is widely distributed throughout the central nervous system (CNS) [9], and serve as both Ca^2+^ transporter and sensor [9–12]. Commonly employed as an neuronal marker in anatomical and developmental studies [13], CB is present in neurons that exhibit selective resistance to neurotoxic insults, such as ischemia, hypoxia and prolonged glutamatergic stimulation [14–16]. In addition to its well-documented anti-apoptotic properties [17–19], CB has also been implicated in region-specific vulnerability to age-related effects [8,20,21]. Indeed, numerous studies have reported an age-dependent decrease in CB immunopositive (+) neurons across cortical and subcortical regions in rodent and primate brains [6,22–31].

Moreover, the CB is closely associated with distinct functional streams within the visual system, particularly those that transverse the dorsal lateral geniculate nucleus (DLG) [32–34]. As the central link between the retina and the visual cortex, the DLG is a thalamic structure involved in re-transmission and modulation of retinal signals to cortical visual areas, thereby contributing to the formation of visual perception [35–37]. In the primate DLG, CB is predominantly expressed in neurons located in the koniocellular (K) layers [32,38,39], a strata that receives signals from the short-wavelength-sensitive cones (S-cones) and mediates blue/yellow color vision [40–43].

Given its critical role in visual processing and potential involvement in age-related visual deficits, the DLG has been scrutinized in aging research, primarily by morphological analysis [44–50]. Classical architectonic staining procedures and neuronal markers, such as the Nissl technique and neuronal nuclei (NeuN), have demonstrated a stability in neuronal number [44,45,49,50] and hypertrophy of neuronal somata in aged DLG [45]. However, these data do not exclude the possibility that CB^+^ neurons within this visual domain may exhibit differential vulnerability to degenerative processes, or that changes in CB expression in the DLG may potentially contribute to the functional decline of the visual capabilities during aging, such as color perception. In fact, it is known that color vision gradually declines with aging [51,52] and there is evidence of impaired discrimination along the blue/yellow opponency axis in aged humans [53,54]. Despite this significance, quantitative assessments of the CB population in aged DLG remains unexplored.

Therefore, given the -potential relationship between aging, Ca_2_^+^ signaling, and visual function, we employed design-based stereological probes to estimate the total number and somal volume of CB^+^ neurons in the DLG at two age groups (adult and aged) in the common marmoset (*Callithrix jacchus*), an emerging non-human primate model for aging research [55,56], to elucidate the age-related morphological changes in a thalamic visual-processing center*.*

## 2. Materials and methods

### 2.1. Animals

Serial brain sections of six marmosets (four adults, two aged, 322–367 g) from the Primatology Center of the Federal University Rio Grande do Norte were used in this study (see Table 1 for details). These animals represent a subset previously described in studies from our laboratory [30,57], with key methodological aspects summarized here. Sex was not used as an exclusion criterion. All experiments were carried out in accordance with Brazilian law number 11.794/2008 governing the ethical use of animals research, and approved by the local animal ethics committee (CEUA-UFRN protocol number 026/2010). Detail of animal husbandry and welfare have been previously published [30,57]. The animals were generally healthy, with no veterinary records of significant or chronic illness, and exhibited no signs of impaired visually guided behavior. Marmosets were considered aged from 96 months old [58].

**Table 1:** Case overview.

Case#	Age (months)	Sex	CB^+^ neuronal number	CB^+^ somal volume (µm^3^)
M6	29	M	7,785	819.40
CNQ_Bl2	36	M	9,421	779.23
J1	39	M	6,854	853.75
M5	65	M	8,095	851.50
Si5	123	F	30,562	804.84
Si4	143	M	15,197	747.04

### 2.2. Tissue acquisition

All animals were pre-medicated with an intramuscular administration of atropine (0.04 mg/kg) and tramadol hydrochloride (5 mg/kg), followed by deep anesthesia induced with ketamine (5 mg/kg) and xylazine (0.5 mg/kg). Subsequently, each animal was transcardially perfused with 400 ml of heparinized saline (0.9%), followed by 700 mL of formalin (10%) in a 0.1 M phosphate buffer (PB, pH 7.4). After perfusion, the brains were extracted, post-fixed in the same medium overnight and cryoprotected in a solution containing 30% sucrose in 0.1 M PB for 3 days. The brains were then snap-frozen and sectioned at 30 μm thickness using a freezing microtome (SM2000 R, Leica Biosystems, Nussloch, Germany). Every sixth section was set aside for CB immunostaining and stored in anti-freezing solution at -20°C.

### 2.3. Region of interest

**Fig 1:**
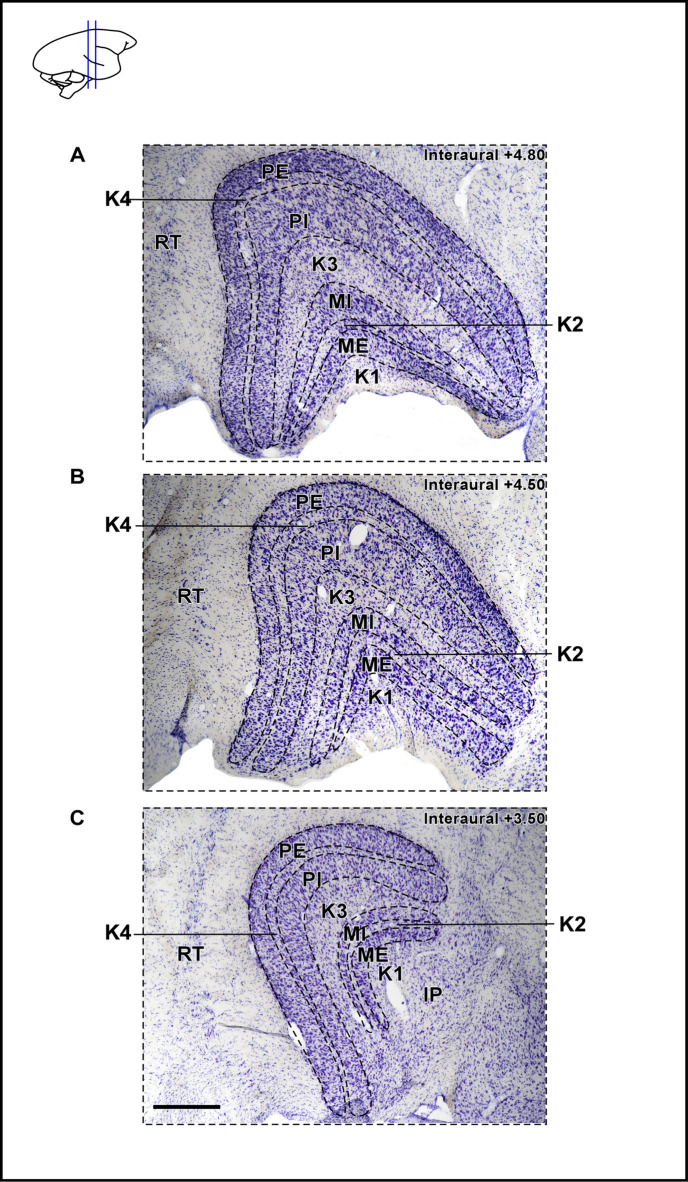
Morphology of marmoset dorsal lateral geniculate nucleus (DLG). (A) Digital photomicrographs of a thionin (Nissl substance) stained coronal sections through DLG, showing external parvocellular (PE), internal parvocellular (PI), internal magnocellular (MI), external magnocellular (ME) and koniocellular (K1–K4) layers. Scale bar: 500 µm. Abbreviations: IP, inferior pulvinar; RT, reticular nucleus.

The DLG represent the primary thalamic division of the mammals visual system, characterized by a layered profile and comprised of three neuronal subtypes, named parvocellular (P), magnocellular (M) and koniocellular (K) cells [59]. In marmoset, the DLG consists of two P layers (internal - PI and external - PE), two M layers (internal - MI and external - ME) and four K laminae (K1–K4), as revealed by Nissl and hematoxylin staining techniques [60, 61, 62]. The laminar structure of DLG is showing in the Fig 1.

### 2.4. Antibody characterization

The antibody used in this study (Sigma-Aldrich, #Cat C9848, RRID: AB_476894) is derived from the CB-955 hybridoma and specifically recognizes CB in several mammal species [6], including the marmoset [63]. Due to its high sensitivity and specificity for detecting CB^+^ neurons across different brain regions, this antibody is widely used as an immunocytochemical marker in neurodevelopmental [64–66] and aging studies [27,30,31], as well as for morphological [67–69] and neuropathological analysis [13,70]. Prior to the immunohistochemical protocol, control DLG sections were processed following the procedures outlined above, with exception of omitting primary antibody. No labelling for CB was observed in control sections.

### 2.5. Immunostaining

Immunohistochemical processing for CB has been described in detail in prior study [30]. Firstly, endogenous peroxidases were inactivated by incubation in a solution containing 0.3% hydrogen peroxide in 0.1 M PB for 20 min. Then, sections were incubated overnight with a monoclonal mouse anti-CB primary antibody (Sigma-Aldrich, #Cat C9848, RRID: AB_476894) in a 1:1,000 dilution containing BSA and 0.1 M PB with 0.4%Triton X-100 (PBTX). After rinsing, sections were incubated with a goat anti-mouse secondary antibody (Jackson ImmunoResearch, Cat# 115-065-003, RRID: AB_2338557) in a1:1.000 dilution with PBTX for 120 minutes. Subsequently, sections were incubated in a 0.4% avidin-biotin solution (Vectastain standard ABC kit, PK-4000, Vector Laboratories), with 2.3% NaCl addiction, for 90 minutes. CB^+^ neurons were then visualized with diaminobenzidine (DAB) working solution combined 0.3% hydrogen peroxide. Importantly, all immunohistochemical reagents used in this study were from the same lot to ensure consistency, and tissue samples from both groups were processed concurrently in the same staining run (side-by-side). Following staining, reactive tissue was mounted onto gelatin-coated glass slides, air dried, dehydrated in graded ethanol series, cleared in xylene, and coverslipped using the neutral mounting medium Entellan® New (Merck, Darmstadt, GER). Additionally, one set of sections was reserved for Nissl (thionin) staining to delineate the architectonic boundaries of the DLG layers. Post-sectioning evaluation revealed a mean reduction of 36.7% in the initial section thickness (t = 30).

### 2.6. Digital photography

All CB^+^ sections were examined using standard light microscopy with a Nikon Eclipse Ni-U microscope (Nikon Corporation, Tokyo, Japan). Photomicrographs from DLG were captured using a digital video camera (Nikon DS-Ri2, Nikon Corporation, Tokyo, Japan) with 5x and 40x objective lenses. All selected images were minimally processed for brightness and contrast before arranging and lettering each panel in specific figures with the software Canvas 12 (ACD Systems, Victoria, Canada, RRID: SCR_014288). The identification of the DLG was based on cytoarchitectonic criteria previously reported [60–62] and aligned with the first edition of the marmoset brain atlas [71].

### 2.7. CB^+^ neuron estimation

For the unbiased stereological approach, the CB^+^ cell number was estimated using the optical fractionator probe, as described by [72]. This analysis was conducted on AxioImager Z2 microscope (Zeiss, Oberkochen, Germany) equipped with a X-Y motorized stage and Z encoder - BX61 motorized stage (Olympus, Tokyo, Japan), and a CX 9000 (MBF Bioscience, Williston, USA) digital camera connected to a computer running the Stereo Investigator 8.1 platform (MBF Bioscience, Williston, USA, RRID: SCR_002526). A 2.5× (0.075 NA) objective lens was used to delineate the DLG (interaural +5.30mm to +3.50mm) contour. Layer-specific quantifications were not performed due to the indistinct boundaries of the K strata. For absolute CB^+^ cell number, counting frames (40 × 40 μm) were systematically distributed at known step length (x, y interval) throughout the DLG, initiated from a random starting point. Each section was sampled with at least 20–25 counting frames using an optical dissector height of 8 μm and a guard zone of 1 μm, as corroborated by our pilot study. Only those CB^+^ neurons exhibiting punctate cellular morphology that were completely within or in contact with the dissector inclusion zone were counted at high magnification (63 × , 1.4 NA) to obtain a minimum of 100 counted cells. Sections were spaced 180 μm apart and sampled using a systematic uniform random paradigm for this analysis. The area sampling fraction (asf) was calculated by dividing the areas of the counting frame by the area of the grid spacing. Additionally, the height sampling fraction (hsf) was determined by dividing the dissector height by the measured section thickness. The total number of counted cells (Q^−^) from the DLG was obtained within a serial sampling fraction (ssf) of 1/6, comprising approximately 8 sections out of a total of 20 DLG sections All sections were blind-coded, and neuronal estimation was conducted bilaterally in each DLG. The mean coefficient of error (CE) for CB^+^ neuronal number for the adult and aged groups was 10.42% and 7.06%, respectively.

The total number of neurons (N) was estimated by the optical fractionator equation [72]:

$$\mathrm{\backslash [}N=\sum Q^{-}\;x\;\frac{1}{hsf}x\;\frac{1}{asf}\;x\;\frac{1}{ssf}\mathrm{\backslash ]}$$

### 2.8. CB^+^somal estimation

The somal volume estimation was conducted as previously described [73]. Briefly, to obtain a systematic random sample of CB^+^ neurons, we performed the nucleator probe [74] simultaneously with the optical fractionator. Cellular volume was estimated for second marked neuron and six rays were used to define the soma boundaries. We used the following equation to estimate the somal volume:

$$\overset{―}{VN}=\;\frac{4\pi }{3}\;\overset{―}{l_{n}^{3}}$$

where “ $\overset{―}{VN}$” is mean volume estimate of all CB^+^ neurons sampled, “ l” is mean all cubic distance from the nucleolus to the cell boundary and “n” equals the number of segments. The mean CE for somal volume were 3.95% and 3.5% for the comparison adult and aged marmosets, respectively.

### 2.9. Statistical analysis

A Shapiro-Wilk test was conducted to assess the normality of the data distribution. The statistical analysis of differences between age groups were determined through permutation tests, using 100,000 sub-sample interactions (see Table 2 for confidence intervals). These statistical analyses were conducted using Phyton version 3.10 and data are presented as mean ± standard deviation. Correlations between age and morpho-quantitative parameters were assessed using Spearman’s test through GraphPad Prism version 10.0 (GraphPad, San Diego, USA, RRID: SCR_002798). Significance was set at the level of 5% (*P* < 0.05).

**Table 2:** Mean, confidence intervals (95%) and observed difference for each factor.

Factor	Mean ± SD (adult)	Mean ± SD (aged)	Observed difference (adult – aged)	Lower bound	Upper bound
CB^+^ neuronal number	8,038.75 ± 1,087.1	22,879.50 ± 10,872.0	‐14,840.75	‐29,713.09	‐6,373.09
CB^+^ somal volume	825.97 ± 33.47	775.94 ± 40.82	50.03	‐14.83	119.40

## 3. Results

### 3.1. CB^+^ neurons are predominantly restrict to K layers

**Fig 2:**
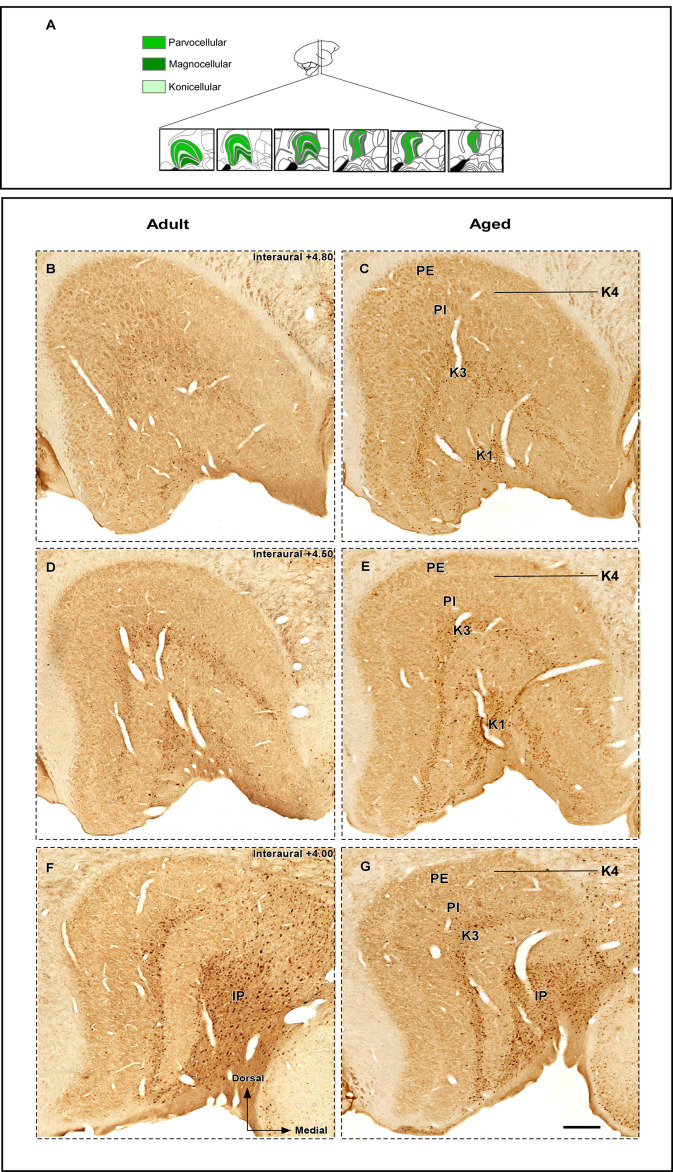
Photomicrographs of CB-immunostained histological sections containing the marmoset dorsal lateral geniculate nucleus (DLG). (A) Representative sections of the DLG used in the present analysis. (B-G) CB immunoreactivity in the DLG of adult (left) and aged (right) animals at rostral (B-C), middle (D-E) and caudal (F-G) levels. Scale bar: 500µm. Abbreviations: IP, inferior pulvinar; K1, koniocellular layer 1; K3, koniocellular layer 3; K4, koniocellular layer 4; PE, external parvocellular layer; PI, internal parvocellular layer; RT, reticular nucleus. Adapted from Paxinos et al. (2012).

Immunostaining for CB was robust with CB^+^ neurons clearly visualized throughout the rostrocaudal extent of the DLG. This immunohistochemical analysis revealed that CB^+^ cells are predominantly confined to the K strata, especially K1 and K3 layers (Fig 2B–G). Scattered CB^+^ neurons were also observed in the M and P layers. However, this technique did not allow for precise delineation of the cytoarchitectonic boundaries of the DLG layers, primarily the K layers adjacent to the inferior pulvinar (Fig 2B–G). Qualitative observations suggest that CB^+^ staining was more intense in aged animals compared to adult ones (Fig 2B–G).

### 3.2. No aging effects in CB^+^ neurons of the DLG

**Fig 3:**
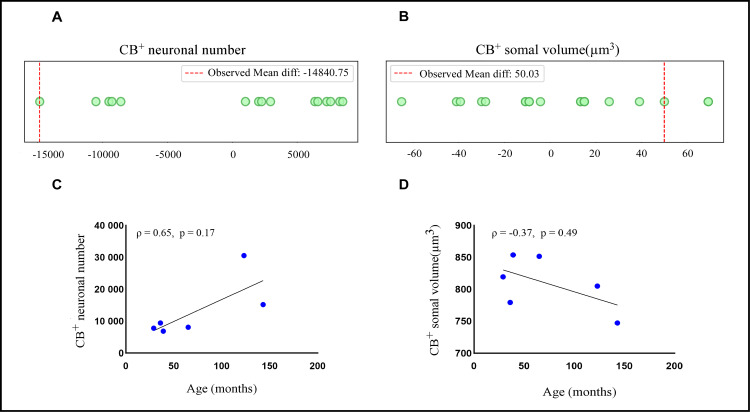
Stereological estimations of morpho-quantitative parameters in marmoset dorsal lateral geniculate nucleus (DLG). (A and B) Dot plot illustrates the distribution and statistical comparison of age groups for (A) CB^+^ neuronal number and (B) CB^+^ somal volume (in µm^3^). The permutation test shows no significant difference for CB^+^ neuronal count (p = 0.06571) and CB^+^ cellular volume (p = 0.19954) between adult (n = 4) and aged animals (n = 2). For this plot, each point represents a mean difference between the permuted data. These findings was further corroborated by (C) Spearman (ρ = 0.65, p = 0.17) and (D) (ρ = -0.37, p = 0.49) correlation tests, which indicated CB^+^ neuronal number and CB^+^ somal volume do not vary significantly with age, respectively. For correlation data, each point represents one individual. Significance at p > 0.05.

The total number of CB^+^ neurons in the DLG (Table 1) was estimated using the optical fractionator method. Qualitative analysis suggested more robust CB^+^ staining in aged animals compared to adult ones (Fig 2B–G). However, this observation was not supported by the permutation test, which revealed no significant difference for CB^+^ neuronal number between age groups (observed mean difference = ‐14840.75, permutated p = 0.06571, observed Cohen’s d = ‐1.92) (Fig 3A). Furthermore, the Spearman’s test demonstrated a positive correlation between CB^+^ cell number and age (Spearman correlation coefficient ρ = 0.65, p = 0.17), albeit it was not statistically significant (Fig 3C).

### 3.3. No age-related changes in CB^+^ somal volume in the DLG

**Fig 4:**
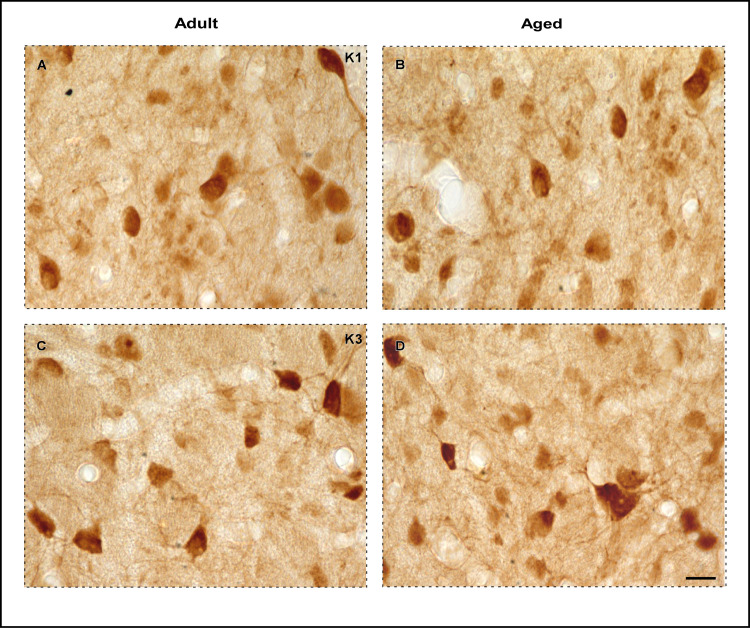
High magnification photomicrographs of CB-immunolabeled DLG neurons. (A–D) CB immunoreactivity in the koniocellular strata of adult (left) and aged (right) animals, specifically in K1 and K3 layers, respectively. Black arrowheads point to neurons with an evident CB immunostaining and punctate cell-like morphology, while white arrowheads indicates cells excluded from the counting due to weak immunolabeling and other morphological shapes. Scale bar: 50µm **(A–D)**.

The volumetric estimation of CB^+^ neuronal soma in the DLG (Table 1) was performed using the nucleator probe. Visually, no differences in somal volume were observed between the age groups (Fig 4). This observation was corroborated by permutation test, which reveals no significant difference in mean somal volume between adult and aged animals (observed mean difference = 50.03, permutated p = 0.19954, observed Cohen’s d = 1.32) (Fig 3B). Additionally, the Spearman correlation analysis revealed a negative, albeit not significant, correlation between somal volume and aging (Spearman correlation coefficient ρ = -0.37, p = 0.49) (Fig 3D).

## 4. Discussion

In the present study, we conducted the first investigation into age-dependent changes in CB morpho-quantitative parameters within the DLG of marmosets aged 29–143 months, employing a design-based stereological approach. Although the immunostaining patterns of CB in the marmoset DLG have been extensively documented [39,58,69] and the neuroprotective effects of CB in pathological degeneration and aging have been well-established [31,75–77], the numerical count and somal volume of the CB^+^ neuronal population in the DLG with aging have not been previously reported. Here, we demonstrated immunohistochemical staining for CB labels neurons located predominantly in the K strata, particularly in the K1 and K3 layers, a data similarly consistent with observations in other primate species [78–80]. Furthermore, our analysis revealed no evidence of morpho-quantitative changes in the CB^+^ cells with aging, marking the first report of aging effects in this neuronal population within the thalamic visual center. This observation was supported by the application of rigorous statistical techniques [81–83] and design-based stereological methods, which offers quantitative structural data without bias related to cellular size, orientation or distributions [72,84]. These findings enhance our understanding of the aging effects on the morphological components of DLG and provide an important foundation for future research on Ca^2+^ homeostasis and differential susceptibility of neuronal subpopulation to dysfunctional or degeneration with aging. Furthermore, the description of age-related changes in CBP expression in visual system may facilitate the development of targeted interventions to mitigate visual decline in the aging population.

### 4.1. CB^+^ neuronal number is stable in the DLG during aging

In this study, we conducted a stereological analysis of the DLG at two distinct age stages across lifespan of marmosets, estimating the CB^+^ neuronal number within this subcortical visual center. Notably, the difficulty in delineating the cytoarchitectonic boundaries of the DLG layers, primarily the K strata at caudal levels, precluded layer-specific estimations. Additionally, the presence of scattered CB^+^ cells within the M and P layers further supports the adoption of an absolute neuronal number analysis. Our findings revealed a stability in the numerical count of CB^+^ neurons with aging, contrasting with the commonly reported age-related decline in CB^+^ neuronal populations across various brain regions [6,22–31]. However, our results are aligned with other studies that have reported numerical stability in the CB^+^ neurons in specific CNS regions during aging, including the *locus coeruleus* [8], retina [85], suprachiasmatic nucleus [86], inferior colliculus [87,88], medial geniculate body, auditory cortex [88], lateral superior olive, medial nucleus of the trapezoid body [84], cochlear nucleus [88,89] and glomerular layer of olfactory bulb [90]. These findings suggest that the marmoset DLG may be less susceptible to the aging effects, specifically in the CB^+^ neuron population, supporting the hypothesis that the vulnerability of CB^+^ neurons to aging is region-dependent. Previous studies in rats and non-human primates have similarly reported a numerical stability in DLG neurons with aging [44,45,49,50,91], and our results extend this observation to the CB^+^ population. This suggests that, in the aged brain, the stability of CB^+^ neurons in the DLG reflects a selective resilience in certain thalamic regions, which contrast with the observed vulnerability in other brain areas.

### 4.2. CB^+^ neuronal number of DLG across sex and dichromatic/trichromatic specimens

Similar to most diurnal Neotropical primates, marmosets exhibit sex-linked polymorphism of color vision [92]. The genes responsible for the expression of photopigments in medium- and long-wavelength-sensitive cones are located at a single X-chromosome locus [93,94]. As result, all males and homozygous females are dichromats, whereas heterozygous females are trichromatics [95,96]. For this reason, it plausible to presume that there are chromatic- and sex-specific variations in the morpho-quantitative parameters of the marmoset DLG. To address this question, [97] assessed the DLG volume and cell number from 10 trichromatic females, 1 dichromatic female, and 13 dichromatic males using model-based stereological approach. These authors demonstrated that males had slightly larger DLG volumes and correspondently more relay cells than females (dichromatic and trichromatics), albeit these differences were not significant. Similarly, dichromatic (pooled data for males and females) had slightly, but not significantly, larger DLG and more relay cells [97]. Thus, these findings evidence negligible differences between male and female marmosets, as well as dichromatic and trichromatic individuals regarding DLG morphology.

Although the present study did not identify the opsin polymorphism phenotype in females or assess sexual differences in marmoset DLG anatomy, it is unlikely that these factors influenced the results obtained, in light of the previous data reported by [97]. We hypothesize that intraspecific variations have contributed to the absence of significant differences between our experimental groups. Interindividual variations were previously reported in morpho-quantitative analysis of the primate DLG during aging. [45], using assumption-based methods, demonstrated that the total number of neurons and neuronal density varied by a factor of 1.9 and approximately 1.7, respectively, in individual young-adult macaque animals [45]. Similarly, intraspecific variations in the volume of the DLG [97–101] and V1 [102], as well as in the number of optic nerve fibers have also been described in both humans and monkeys [103,104], as revealed in aging and anatomical studies. Therefore, we propose that high intraspecific variability could also account for the absence of significant differences between our experimental groups (see comments in topic 4.6). However, whether this variability in the morpho-quantitative parameters of the primate DLG affects visual processing will require further analysis.

### 4.3. Preserved CB^+^ cellular volume in the DLG during aging

Cytological parameters, such as somal volume, provide valuable insights into the structural integrity of neurons in the aged brain [105]. In this study, we focused on estimating the cellular volume of CB^+^ neurons in the marmoset DLG during aging using the nucleator probe. Our findings indicate that the somal volume of CB^+^ neurons remains preserved with aging, contributing to the limited but growing body of knowledge on cytological parameters associated with neuronal aging. Although a substantial body of research has been centered on the numerical changes in CB^+^ neurons with aging, fewer studies have investigated alterations in cellular volume or soma size, making our findings particularly relevant to understanding the morphological stability of CB^+^ neurons. In different rat strains, [20] observed a significant decrease in the average volume of CB^+^ neuronal somas with aging in the dorsal cortex of the inferior colliculus and ventral subdivision of the medial geniculate body, based on cross-sectional area assessment. Conversely, [87] reported that the area of CB^+^ neurons soma is unaltered with aging in the inferior colliculus, medial geniculate body, auditory cortex, superior olivary and cochlear nuclei of the aged rats. These discrepancies in the literature could attributed to methodological differences, such as the techniques used for assessing neuronal volume, while they might also reflect regional and species-specific variations in how neuronal structures respond to aging. Our study contributes to the existing literature by demonstrating that, at least in the marmoset DLG, the somal volume of CB^+^ neurons remains stable across the lifespan.

### 4.4. Does CB serves as a biological marker for age-related changes of cytological parameters in the DLG?

The examination of cytological parameters in aged DLG has yielded a plethora of findings that are, at times, contradictory across various species and methodologies [45,46,48,106]. The morphological changes reported in these studies, such as variations in neuronal diameter, area, or somal volume, are frequently based on classical histological techniques, such as Nissl or toluidine blue staining [45,46,48,106]. However, these methods do not distinguish specific neuronal subpopulations, making it challenge to assess the cell-specific effects of aging. [45] observed an increase in neuronal area in the P and M layers of the macaque DLG with aging, whereas studies in aged rats reported conflicting results. [106] found a slight reduction in neuronal diameter, while [46] and [48] documented an increase in neuronal size and a decline in somal volume, respectively. These discrepancies may originate from the use of two-dimensional quantitative techniques and the reliance on non-specific histological stains, which do to account for potential variations in neuronal types or their susceptibility to the aging effects.

In contrast, our study offers a novel perspective by employing a design-based stereological approach combined with immunohistochemistry for CB to specifically target CB^+^ neurons in the DLG. These combined method allows for an unbiased, three-dimensional assessments of neuronal volume and number, avoiding the limitations of earlier two-dimensional studies. However, it should be noted that our analysis for neuronal volume is not equivalent to the study of three-dimensionally reconstructed neurons from serial imaging due a methodological constraints (see section 4.6).

### 4.5. Functional implications

In this study, we also explored whether age-related visual deficits, such as a decline in blue/yellow color discrimination, could be associated with morphological changes in CB^+^ neurons of the DLG. Although our results did not reveal anatomical evidence to directly support this visual decline, the absence of significant findings regarding CB^+^ neurons does not preclude the potential involvement of other mechanisms associated with Ca^2+^ dysregulation in age-related visual impairments [8]. First, CB belongs to the CBPs family that regulate intracellular Ca^2+^ levels [9,107], and changes in the expression of other CBPs with aging could contribute to DLG susceptibility to dysfunctional state. For example, previous studies have shown an increased density of parvalbumin-expressing neurons in the M and P layers of aged macaques [108], suggesting that other CBPs may respond differently to aging. Conversely, an age-dependent loss of calretinin-expressing neurons has been reported in DLG rats [109]. Second, CBPs constitute merely one component of the extensive network by which neurons regulate intracellular Ca^2+^ levels [2,110]. Others important factors, such as Ca^2+^ pumps, stores, sequestering agents, and channels, also play a critical role in the maintenance of Ca^2+^ homeostasis within neurons, and age-related changes in these components may lead to DLG dysfunction through Ca^2+^ excitotoxicity[8]. Further investigation is required to ascertain the potential neuroprotective role of CB and other CBPs in the DLG, as well as their contributions to the preservation of visual function with aging. Understanding these mechanisms could have significant implications for addressing age-related visual impairments and may offer valuable insights for the development of neuroprotective strategies that target Ca^2+^ homeostasis.

### 4.6. Limitations

The morpho-quantitative parameters reported in the present study should be considered with certain limitations. First, the analysis of nervous tissue samples was performed using a small n-size design (n_total_ = 6) due to the difficulty in obtaining aged marmoset, i.e., animals aged from 96 months old [58], since most experimental marmoset colonies (such as our primatology center) have a median lifespan of 60–72 months [55]. This further constrained our sample size, since we used aged animals with an average age of 133 months. Although this limitation may affect the quality of the statistical model, this sample size has been employed in other studies investigating the aging effects on brain morphology [50]. Furthermore, to address the limitation posed by the small sample size, we also employed correlation analyses to examine potential linear relationships between aging and morpho-quantitative parameters assessed. Additionally, we used a combination of traditional non-parametric analysis and permutation-based statistical procedures, which the latter serves as robust re-sampling alternative especially useful when large-sample approximations are not feasible [111,112]. Nevertheless, in future studies, it will be crucial to use large sample size to conduct a more comprehensive exploration of how aging impact the morphological parameters of the visual system, particularly the CB^+^ neuronal subpopulation.

Second, the estimations obtained in this study were limited by the use of coronal sections, which hindered the application of completely unbiased techniques for somal volumes estimation, as decribed by [73]. In line with those observations, our study faced similar constraints, albeit they did not impact the measurements of the total number of neurons, which remain independent of orientation [84] and constitute the primary variable of interest. Consequently, our estimates of somal volume are constrained by bias due to the absence of random rotation during tissue processing. The magnitude of this bias is contingent upon the degree of anisotropy in both shape and orientation of DLG neurons and it is no possible determined from the material in this work, albeit it is evidently less significant compared to more anisotropic brain territories, such as cerebral cortex [84]. Importantly, stereologically appropriate randomly rotated sections in the thalamus pose a major challenge to obtaining accurate identifications in all thalamic regions, including the DLG [see 84 for comments]. Although the method proposed in a previous work  [113] may address this issue, its implementation was not feasible due to the initial tissue preparation procedures adopted in our study. In addition, the coronal slice plane was also previously used in for evaluating somal volume in primate brains [73,114,115]. Therefore, unbiased stereological studies are needed to corroborate our findings in terms of neuronal volume for the DLG CB^+^ subpopulation.

Finally, age-related shrinkage and tissue distortion introduced by the tissue processing were not monitored in the present study. Although these deformational changes are recognized biases in morphometric quantifications [116,117], they are resolved in cell number estimation, since the optical fractionator probe is not affected by volumetric changes and relies on a relative measures for sampling the tissue thickness [118]. However, local volume probes, such as the nucleator, can be influenced by these biases, affecting the validity of the cellular volume estimation [119]. Notably, while fixation-related shrinkage is a relevant factor in volumetric estimations [120], it is not considered the primary cause of shrinkage [99], and it has been reported to induce approximately less than 3% shrinkage [121]. Shrinkage due to tissue processing/embedding is the main source of shrinkage and was the parameter described as affected by aging [116]. Although the nucleator probe is not formulated around the section-cut thickness, the volumetric data we provide here are also limited in the sense that it is not controlled for the fixation and processing-related differential thickness.

Addressing tissue shrinkage is a complex problem to solve and predict, with several proposed methods for mitigating its effects, including those by [119] and [120], who recommended glycol methacrylate embedding to minimizes tissue deformation. Nevertheless, these methods are challenging compared to routine processing, which requires specialized microtomes for sectioning and modifications to staining techniques that are difficult to implement [115]. Although these factors affect the analysis of marmoset material, our study adhered to the recommendations of [119] and [120] for cellular volume, performing z-axis measurements, and using mounted frozen sections to reduce distortion.

## 5. Conclusions

In this study, our design-based stereological approach did not reveal age-induced morphological quantitative changes in CB^+^ cells of the marmoset DLG in terms of neuronal number and somal volume. This structural stability suggests that the CB^+^ neurons in aged DLG may be less susceptible to Ca^2+^-mediated dysfunctional and neurodegenerative processes. Although we did not investigate the functional significance of the observed stability of CB^+^ neurons in aged DLG, the morphological data described here provides a valuable resource for future studies exploring the neuroprotective role CB play in visual processing during physiological aging. Furthermore, our findings contribute to the development of neuroprotective strategies specifically tailored to vulnerable neuronal populations by demonstrating the preservation of the morphological aspects of CB^+^ cells in the DLG with aging.
